# Early clinical predictors for the prognosis of invasive pneumococcal disease

**DOI:** 10.1186/s12879-020-05382-z

**Published:** 2020-09-04

**Authors:** Shuiyan Wu, Xubei Guo, Zhong Xu, Meilin Han, Lili Huang, Yunzhen Tao, Ying Li, Yanhong Li, Tao Zhang, Zhenjiang Bai

**Affiliations:** 1 Pediatric Intensive Care Unit, Children’s Hospital of Soochow University, Suzhou, Jiangsu China; 2 Laboratory department, Children’s Hospital of Soochow University, Suzhou, Jiangsu China; 3 Nephrology department, Children’s Hospital of Soochow University, Suzhou, Jiangsu China; 4 Key Laboratory of Public Health Safety, Ministry of Education-department of Epidemiology, School of Public Health, Fudan University, Shanghai, China

**Keywords:** Children, Invasive pneumococcal disease, Mortality, Risk factors, Serotype

## Abstract

**Background:**

Risk factors related to mortality due to invasive pneumococcal disease (IPD) have been unveiled previously, but early clinical manifestations of IPD based on prognosis remain uncovered.

**Methods:**

The demographic characteristics, clinical features, serotype, antibiotic susceptibility, and outcomes of 97 hospitalized children with laboratory-confirmed IPD from Suzhou, China, were collected and analyzed retrospectively.

**Results:**

The median age was 0.69 (0.49–1.55) years in the non-survivor group compared with 2.39 (0.90–3.81) years in the survivor group. The mortality of 97 children with laboratory-confirmed IPD was 17.5% (17/97), and 53.6% of them were aged less than 2 years. Pathogens were mainly from the blood and cerebrospinal fluid, and sepsis was the most frequent type. Statistically significant differences were found in hyperpyrexia, vomiting, anorexia, lethargy, poor perfusion of extremities, Hb level, and Plt count between the nonsurvival and survival groups. Further, the multivariate regression analysis showed that early signs, including hyperpyrexia, vomiting, anorexia, lethargy, and poor perfusion of extremities, were independent risk factors for the in-hospital mortality of children with laboratory-confirmed IPD. The mortality was also associated with antimicrobial sensitivity in pneumococcal isolates. The microbes in 1/17 (5.9%) children who were prescribed an antibiotic showed antimicrobial sensitivity in the nonsurvival group, compared with 21/80 (26.3%) children who survived. The most common serotypes identified were 6B (35.3%, 6/17), 14 (23.5%, 4/17), 19F (23.5%, 4/17), 19A (5.9%, 1/17), 23F (5.9%, 1/17), and 20 (5.9%, 1/17) in the nonsurvival group. The coverage of IPD serotypes of the 7-valent pneumococcal conjugate vaccine (PCV7) was 88.2% (15/17), while that of the 13-valent *S. pneumoniae* vaccine (PCV13) was 94.1% (16/17) of the coverage in the nonsurvival group.

**Conclusions:**

Recurrent hyperpyrexia, vomiting, anorexia, lethargy, and poor perfusion of extremities in the early stage were independent predictors for the in-hospital mortality of children with laboratory-confirmed IPD. Appropriate use of antibiotics and PCV immunization were the keys to improve the outcome of IPD.

## Background

*Streptococcus pneumoniae* (*S. pneumoniae*) infections are the leading cause of death from a vaccine-preventable illness in children aged less than 5 years, accounting for 18.3% of severe pneumonia and 33% of death caused by pneumonia [1]. *S. pneumoniae* can also cause invasive pneumococcal disease (IPD), including pneumonia, meningitis, osteomyelitis, and sepsis [2]. IPD is defined as a disease in which *S. pneumoniae* is cultured from normal sterile sites, such as blood, cerebrospinal fluid, pleural effusion, biopsy, joint effusion, or peritoneal effusion [3]. Previous reports found that pneumococcal conjugate vaccine (PCV) immunization could effectively prevent IPD [2, 4, 5]. Therefore, vaccine immunization is an excellent strategy to reduce the incidence and mortality of IPD.

However, PCV immunization is not considered a routine vaccination by the government in many countries, especially China. Hence, the incidence and mortality are still high in these countries. Although Suzhou is one of the wealthiest cities in China, only 2–10% of patients were vaccinated during the study period. Many studies identified some features of in-hospital IPD in adults and children [6–9], but the initial clinical characteristics for prognosis were not reported. The symptoms and signs of IPD are related to many factors such as host immunity, *S. pneumoniae* virulence, and site of infection [10]. Early recognition and prompt diagnosis remain the challenges. The present study was performed to analyze the clinical features and outcomes of children, so as to find a better strategy for reducing the incidence and mortality of IPD within 24 h of the onset in China.

## Methods

### Clinical data collection

All children admitted to the pediatric intensive care unit of Children’s Hospital of Soochow University with the isolation of *S. pneumoniae* from sterile sites (blood, pleural fluid, and cerebrospinal fluid) were included from January 2011 to December 2017. This study cohort comprised children aged 0.16–11.26 years. None of them received a pneumococcal vaccine. Data of patients, including demographics, symptoms and signs, laboratory data, and empiric and definite antimicrobial agents within 24 h of the onset of the disease, were collected from the clinical records retrospectively. The patients were divided into drug-sensitivity-consistent and drug-sensitivity-inconsistent groups depending on whether the antibiotic use was consistent with drug sensitivity. The details on the final diagnosis and outcome were also collected. All patients were divided into survival and nonsurvival (death) groups according to their survival within 30 days. The study procedures were conducted in accordance with the declaration of Helsinki. Written informed consent was obtained from the family involved. The study was reviewed by the Institutional Review Board in the hospital (ethics approval number: 2020CS069).

### Definitions

The diagnosis of bacterial meningitis was according to the criteria of World Health Organization case definition [11]: (1) sudden onset of fever (> 38.5 °C rectal or > 38.0 °C axillary); (2) one of the following symptoms or signs: headache, altered consciousness, or meningeal irritation; (3) cerebrospinal fluid examination showing either of the following: leukocytosis (> 100 × 10^6^ cells/L) or leukocytosis (10–100 × 10^6^ cells/L) with an elevated protein (> 100 mg/dL) or decreased glucose (< 40 mg/dL); and (4) positive culture, positive Gram stain, or positive bacterial antigen in the CSF. Patients who met diagnostic criteria 1, 2, 3, and 4 were considered confirmed cases. The diagnosis of sepsis was according to the diagnostic criteria of “Surviving Sepsis Campaign: international guidelines for the management of severe sepsis and septic shock, 2012” [4]: fever (anal temperature > 38.5 °C) or hypothermia (anal temperature < 35 °C), tachycardia, and at least one of the following complications: consciousness change, hypoxemia, or elevated serum lactate levels. Shock, DIC, respiratory failure, poor perfusion of extremities, and acute kidney injury (AKI) were diagnosed by physicians according to the criteria described previously [4, 12, 13].

### Specimen acquisition and strain isolation

Clinical specimens were collected at admission, including blood, cerebrospinal fluid, and pleural effusion. The cerebrospinal fluid was collected from children with suspected central infections, and pleural effusion was collected from children with pleural effusion. Once the specimen was obtained, it was immediately sent to the microbiology laboratory. Bacterial isolation and culture were carried out according to the “National Clinical Laboratory Procedures” for clinical microbiological testing [14]. The obtained bacteria were subjected to Gram staining, and the morphology of the bacteria was observed. The optochin sensitivity test was used, and the bile solubility test was used as an auxiliary identification test.

### Drug susceptibility test

The bacteria in the logarithmic growth phase after isolating colonies were adjusted. The optical density value was adjusted to 0.5 McFarland Standard with physiological saline, and the minimal inhibitory concentration value was determined according to the operational instructions of the *S. pneumoniae* susceptibility kit (Biomerieux, France) [14]. The size of the rifampicin and linezolid inhibition zone was measured by the Kirby–Bauer disk diffusion method. The size of the inhibition zone was interpreted in accordance with the CLSI2012 standard. The quality control was *S. pneumoniae* ATCC49619, which was purchased from the Clinical Testing Center of the Ministry of Health of China.

### Serotyping of *S. pneumoniae*

The serotypes of all isolates were typed with a capsule-quelling test using type-specific antisera (Statens Serum Institute, Copenhagen, Denmark). The experimental procedure was carried out as previously described [15].

### Statistical analysis

Data were analyzed using SPSS 22.0 software. The normal-distribution measurement data were expressed as mean ± standard deviation (x ± s). The two groups were compared using the *t* test, *χ*^2^ test, or exact probability method. The nonnormal-distribution data were expressed by the quartile method and compared using the Mann–Whitney *U* test. A *P*-value of less than 0.05 was considered statistically significant.

## Results

### Demographics

The median age of 97 children with laboratory-confirmed IPD was 1.88 (0.78–3.64) years. The mortality rate was 17.5% (17/97); 91.8% (89/97) were aged less than 5 years, and 53.6% (52/97) were aged less than 2 years. The nonsurvival group (*n* = 17) comprised 100% (17/17) aged less than 5 years and 82.4% (14/17) aged less than 2 years. The median age of the patients in the nonsurvival group was 0.69 (0.49–1.55) years, significantly less than 2.39 (0.90–3.81) years in the survival group (*n* = 80) (*P* = 0.001). The median age of patients in the meningitis group (*n* = 46) was 0.99 (0.64–2.35) years, significantly less than 2.78 (1.09–3.52) years in the non-meningitis group (*n* = 51) (*P* = 0.031). The median age was 1.54 (0.76–3.44) years in the sepsis group (*n* = 86) and 1.36 (0.73–2.31) years in the non-sepsis group (*n* = 11) (*P =* 0.065).

### Clinical manifestations

Among the 97 specimens, blood culture specimens accounted for 61 patients (61/97, 62.9%), cerebrospinal fluid culture specimens for 27 patients (27/97, 27.8%), and pleural effusion culture specimens for 9 patients (9/97, 9.3%). Further, 42 patients had sepsis alone, 35 had sepsis with meningitis, 9 had sepsis with severe pneumonia, 1 had sepsis with osteomyelitis, and 11 had meningitis alone.

The incidence rates of hyperpyrexia (temperature more than 40 °C), vomiting (greater than three times a day), anorexia (50% less than usual), lethargy, and poor perfusion of extremities among nonsurvivors compared with survivors were 41% vs 12, 70% vs 30, 94% vs 60, 94% vs 36, and 76% vs 12%, respectively, which were significantly higher in the nonsurvival group than in the survival group within 24 h of the onset of the disease (Table 1). Hemoglobin (Hb), platelet (Plt) count, white blood cell count, and neutropenia levels were 94.29 ± 25.74, 215.07 ± 158.07, 8.45 ± 8.42 and 6.10 ± 6.62 in the nonsurvival group, and 110.42 ± 19.23, 352.06 ± 164.66, 19.74 ± 22.67, and 66.67 ± 19.26 in the survival group. The nonsurvival group had significantly lower hemoglobin (Hb) level and Plt count compared with the survival group. However, the C-reactive protein level was not statistically different between the two groups (*P* = 0.968) (Table 1).

**Table 1:** Comparison of clinical manifestations between non-survivors and survivors of children with IPD

Clinical information	Death, *n* = 17	Survival, *n* = 80	*P*
Age(y)	0.69 (0.49, 1.55)	2.39 (0.90, 3.81)	0.001
Male	11 (64.7%)	45 (56.2%)	0.522
PRISMIII score	21.04 ± 10.09	6.78 ± 12.32	0.000
Symptoms and signs			
Hyperpyrexia	7 (41.2%)	10 (12.5%)	0.010^*^
Vomiting	12 (70.6%)	24 (30.0%)	0.002
Anorexia	16 (94.1%)	48 (60.0%)	0.007
Lethargy	16 (94.1%)	29 (36.3%)	0.000
Oliguria	4 (23.5%)	12 (15.0%)	0.295^*^
Poor perfusion of extremities	13 (76.5%)	10 (12.5%)	0.000^*^
Disease category			0.000
Sepsis	0	42	
Sepsis with meningitis	17	18	
Meningitis alone	0	11	
Sepsis with pneumonia	0	9	
Source of pathogens			0.083
Blood	9	52	
Cerebrospinal fluid	8	19	
Pleural effusion	0	9	
Laboratory results			
Hb(g/L)	94.29 ± 25.74	110.42 ±19.23	0.006
WBC(× 10^9^ /L)	8.45 ± 8.42	19.74 ± 22.67	0.071
N#(× 10^9^ /L)	6.10 ±6.62	66.67 ± 19.26	0.052
L#(× 10^9^ /L)	29.48 ± 17.17	24.91 ± 17.34	0.478
Plt(× 10^9^ /L)	215.07 ± 158.07	352.06 ± 164.66	0.005
CRP (mg/L)	40.20 ± 66.69	47.42 ± 69.43	0.968

Hyperpyrexia: fever over 40 °C; vomiting: greater than 3 times/d; anorexia: 50% less than of usual; *Hb* hemoglobin, *N#* neutrophil absolute value, *L#* lymphacyte absolute value, *CRP* C-reactive protein

*Calculated using Fisher’s exact test

The multivariate regression analysis showed that hyperpyrexia, vomiting, lethargy, and poor perfusion of extremities were independent risk factors for the in-hospital mortality of children with IPD (Table 2). The pediatric risk of mortality III (PRISM III) score was significantly higher in the nonsurvival group than in the survival group (Table 2). Shock, respiratory failure, AKI, and convulsions or coma in the early stage were rare, and therefore statistical analysis was not done. Two patients died and two were alive with shock, three patients died and one was alive with respiratory failure, and two patients died and one was alive with seizure. Only one patient showed AKI in the nonsurvival group.

**Table 2:** Multiple logistic regression analyses of early clinical manifestations potentially associated with in-hospital mortality

	Multivariate logistic regression	
	OR(95%CI)	*P* value
Age(y)	1.649 (0.439, 1.187)	0.199^a^
PRISMIII score	7.591 (1.028, 1.179)	0.006^b^
Hyperpyrexia	5.372 (1.294, 21.710)	0.020^c^
Vomiting	6.013 (1.023, 26.522)	0.014^c^
Lethargy	9.633 (3.166, 164.297)	0.002^c^
Poor perfusion of extremities	8.348 (1.910, 36.485)	0.001^c^

Hyperpyrexia: fever over 40 °C; vomiting: greater than 3 times/d; *CI* confidence interval, *OR* odds ratio

^a^after adjustment for PRISMIII score

^b^after adjustment for age

^c^after adjustment for age and PRISMIII score

### Selection of antibiotics

The frequency of antibiotic use was as follows: azithromycin 30.2% (19/63), cefazolin 17.5% (11/63), oxycephalosporin 15.9% (10/63), ceftriaxone 12.7% (8/63), cefonicid 11.1% (7/63), cefodizime 9.5% (6/63), ceftizoxime 9.5% (6/63), cefoxitin 7.9% (5/63), and cefuroxime 6.3% (4/63). The consistency between the choice of antibiotics and the drug sensitivity test was 22.7% (22/97). The rationality of antibiotic choice was 5.9% in the nonsurvival group, which was lower than 26.3% in the survival group (*P* = 0.050) (Table 3). Besides, a comparison of the percentage of antibiotic change in survivors and nonsurvivors showed a high frequency of antibiotic replacement (88% vs 94%, *P* = 0.261). The outcome was not related to the use of antibiotics orally or intravenously. The resistance rate in 97 cases of S. pneumoniae was 100% (97/97) to erythromycin, 97.9% (95/97) to clindamycin, 87.6% (85/97) to tetracycline, 72.2% (70/97) to sulfamethoxazole, 49.5% (48/97) to penicillin, 33.0% (32/97) to cefotaxime, 8.2% (8/97) to amoxicillin, 5.2% (5/97) to chloramphenicol, 0.0% (0/80) to vancomycin, and 0.0% (0/80) to levofloxacin.

**Table 3:** Comparison of rationality of antibiotic choice between non-survivors and survivors of children with IPD

Events about antibiotic choice	Death	Survival	*P*
whether used antibiotics	12/17 (70.6%)	52/80 (65.0%)	0.288
whether used antibiotics intravenously	10/14 (71.4%)	45/80 (56.3%)	0.840^*^
whether the choice of antibiotics is consistent with drug susceptibility	1/17 (5.9%)	21/80 (26.3%)	0.050^*^
percentage of antibiotic change	15/17 (88.2%)	75/80 (93.8%)	0.261

*Calculated using Fisher’s exact test

### Serotype analysis

Ten serotypes of *S. pneumoniae* were detected in 97 hospitalized children with laboratory-confirmed IPD, followed by 6B (24.7%, 24/97), 14 (20.6%, 20/97), 19F (18.6%, 18/97), 19A (15.5%, 15/97), 23F (7.2%, 7/97), 9 V (5.2%, 5/97), 20 (4.1%, 4/97), 15B/C (2.1%, 2/97), 6A (1.0%, 1/97), and 4 (1.0%, 1/97). However, six serotypes including 6B (35.3%, 6/17), 14 (23.5%, 4/17), 19F (23.5%, 4/17), 19A (5.9%, 1/17), 23F (5.9%, 1/17), and 20 (5.9%, 1/17) in nonsurvivors. No significant correlation was found between serotypes and vomiting, anorexia, drowsiness and shock, respiratory failure, AKI, convulsion, and coma. Moreover, no significant correlation was observed between serotypes and mortality and PRISMIII score.

Further, none of the 97 children with laboratory-confirmed IPD received a pneumococcal vaccine. The 7-valent PCV (PCV7) covered 77.3% (75/97) of the IPD serotype, which was significantly lower than 93.8% (91/97) of the 13-valent PCV (PCV13). The 7-valent *S. pneumoniae* vaccine (PCV7) covered 88.2% (15/17) of nonsurvivors, while 13-valent *S. pneumoniae* vaccine (PCV13) covered 94.1% (16/17) (Table 4).

**Table 4:** Serotype distribution analysis between the two groups

	Death, *n* = 17	Survival, *n* = 80	Total
PCV7 serptypes			75
6B	6	18	24
14	4	16	20
19F	4	14	18
23F	1	6	7
4	0	1	1
9 V	0	5	5
PCV13 serotypes not included in PCV7			16
6A	0	1	1
19A	1	14	15
3	0	0	0
Others			6
20	1	3	4
15B/C	0	2	2

## Discussion

In this study, the median age of hospitalized children with laboratory-confirmed IPD was only 1.88 (0.78–3.64) years, accounting for 53.6% aged less than 2 years. The median age of 17 nonsurvivors was only 0.69 years, accounting for 82.4% aged less than 2 years. According to the reports on high-income countries, the annual incidence of IPD in children aged less than 2 years was 160/100,000 [16], indicating that these children were at high risk of *S. pneumoniae* infection. Additional studies showed *S. pneumoniae* infection in infants from Papua New Guinea and Australia [17, 18]. Moreover, approximately half of children from Sweden and the United States were infected with *S. pneumoniae* at least once before the age of 2 years [19, 20]; these results were the same as the present findings. The predisposing factors of this population were not fully understood. It was speculated that the infection might be related to the immature immune system of infants and young children [21]. Therefore, it is recommended internationally to inoculate a four-time vaccination strategy of the PCV13 for individuals aged 2, 4, 6, and 12–15 months, and before the age of 2 years [5].

Furthermore, children who died of IPD showed more nonspecific symptoms, such as recurrent hyperpyrexia, vomiting, lethargy, and poor perfusion of extremities in the early stage. Some vigilance and close observation of children with nonspecific symptoms in the early stage are required to deal with the progression of the disease. This study found that 43.7% (45/97) patients had a change in consciousness during admission, which was higher than that in 11.5% of other patients with pneumonia [22]. All of the nonsurvivors were diagnosed with sepsis with meningitis, and therefore these signs might indicate increased intracranial pressure (IICP). *S. pneumoniae* results not only in disruption of the blood–brain barrier but also in vascular and neuronal injury, finally leading to IICP [22, 23]. Except for early antibiotic use, aggressive control of IICP is also important [24]. Previous studies showed that corticosteroid treatment reduced the death rate of patients with meningitis and *S. pneumonia* infection [25]. Therefore, close monitoring of the child’s state is of great significance for the timely and effective treatment and prognosis of IPD.

The initial consistency rate between antibiotic selection and drug sensitivity was only 22.7%. The rationality of antibiotic choice in the survival group was 26.3%, which was about four times that in the nonsurvival group. It also suggested a trend toward the impact of a reasonable selection of early antibiotics on mortality. In the present study, the resistance rate of *S. pneumoniae* was 100% to erythromycin, 49.5% to penicillin, 33.0% to cefotaxime, 8.2% to amoxicillin, and 0% to vancomycin or levofloxacin. The strains of nonsurvivors in this study were multi-drug resistant. Domestic and foreign reports also showed that *S. pneumoniae* was generally multi-drug resistant, and the resistance to penicillin and cephalosporins increased [6, 26, 27]. Compared with drug sensitivity analysis in the present study, a better consistency rate was achieved if the choice of antibiotics strictly abided by China’s “Guidelines for the Management of Community Communicable Pneumonia (2013 Revision)”. The results suggested that physicians should be encouraged to follow a standard protocol based on the local patterns of antimicrobial sensitivity for community-acquired infection with modifications in the hospital as needed following the documentation of antimicrobial resistance patterns [28].

Ten serotypes of *S. pneumoniae* were detected in 97 hospitalized children with laboratory-confirmed IPD and 6 serotypes in nonsurvivors; 6B was the leading serotype, followed by 14, 19F, 19A, and 23F, which were similar to the top 5 serotypes in the other cities in China, but the sequence was different. The serotypes of IPD cases in the multicenter study of Chinese hospitals were 19F, 14, 19A, 6B, and 23F [29, 30]. In this study, the PCV13 could cover 93.8% of serotypes of IPD cases and 94.1% of nonsurvivors. A large number of studies also showed that PCV13 had a good coverage for the serotypes of IPD. The widespread vaccination of PCV13 in many countries has effectively reduced the incidence and mortality of IPD [31, 32]. At present, China’s PCV13 has not been included in the first-line vaccination catalog. Moreover, this vaccine is expensive (800 RMB/dose, approximately 114 $/dose). Therefore, very few children receive the PCV13 vaccine. About 2–10% of patients were vaccinated during the study period in Suzhou. The high coverage rate of PCV13 indicated that PCV13 could prevent pneumococcal diseases effectively. Unfortunately, none of the children with IPD were vaccinated with PCV13 or PCV7 vaccine in this study. Therefore, PCV13 should be underscored as an agent for preventable mortality in young children (particularly those less than 2 years of age), and thus should be adopted as part of the routine immunization schedule in China.

The present study had several limitations. First, the data of illness onset and presentation to hospital between survivors and nonsurvivors were not specified. So, their influence on the results could not be ruled out. Second, this was a retrospective, single-center study, and the sample size was limited. These factors reduced the strength of the findings. In addition, due to false-negative culture results, some true IPD cases were not included. Next-generation sequencing might improve the diagnostic yield. More large-scale, comprehensive clinical studies and highly sensitive techniques are needed to confirm the conclusions.

## Conclusions

In short, a majority of nonsurvivors with laboratory-confirmed IPD were always younger than 2 years. Early signs, including recurrent hyperpyrexia, vomiting, lethargy, and poor perfusion of extremities, were the poor predictors of the outcome of laboratory-confirmed IPD. Inappropriate use of antibiotics for invasive pneumococcal infection and the low rate of PCV immunization in China were responsible for the high mortality of laboratory-confirmed IPD.

## Data Availability

The datasets used and/or analysed during the current study are available from the corresponding author on reasonable request.

## Abbreviations

SP: *Streptococcus pneumoniae*
IPD: Invasive pneumococcal disease
PCV: Pneumococcal conjugate vaccine

## References

[1] Walker CLF, Rudan I, Liu L, Nair H, Theodoratou E, Bhutta ZA, O'Brien KL, Campbell H, Black RE (2013). Global burden of childhood pneumonia and diarrhoea. Lancet.

[2] Prevention of pneumococcal disease: recommendations of the Advisory Committee on Immunization Practices (ACIP). MMWR Recomm Rep. 1997;46(RR-8):1–24. 9132580

[3] Marrie TJ, Tyrrell GJ, Majumdar SR, Eurich DT (2017). Invasive pneumococcal disease: still lots to learn and a need for standardized data collection instruments. Can Respir J.

[4] Dellinger RP, Levy MM, Rhodes A, Annane D, Gerlach H, Opal SM, Sevransky JE, Sprung CL, Douglas IS, Jaeschke R (2013). Surviving Sepsis campaign: international guidelines for management of severe sepsis and septic shock, 2012. Intensive Care Med.

[5] Nuorti JP, Whitney CG, Centers for Disease C, Prevention (2010). Prevention of pneumococcal disease among infants and children - use of 13-valent pneumococcal conjugate vaccine and 23-valent pneumococcal polysaccharide vaccine - recommendations of the Advisory Committee on Immunization Practices (ACIP). MMWR Recomm Rep.

[6] Ding Y, Geng Q, Tao Y, Lin Y, Wang Y, Black S, Zhao G, Zhang T (2015). Etiology and epidemiology of children with acute otitis media and spontaneous otorrhea in Suzhou, China. Pediatr Infect Dis J.

[7] Hausdorff WP, Bryant J, Paradiso PR, Siber GR (2000). Which pneumococcal serogroups cause the most invasive disease: implications for conjugate vaccine formulation and use, part I. Clin Infect Dis.

[8] Rudan I, Chan KY, Zhang JS, Theodoratou E, Feng XL, Salomon JA, Lawn JE, Cousens S, Black RE, Guo Y (2010). Causes of deaths in children younger than 5 years in China in 2008. Lancet.

[9] Martinez-Vega R, Jauneikaite E, Thoon KC, Chua HY, Huishi Chua A, Khong WX, Tan BH, Low Guek Hong J, Venkatachalam I, Anantharajah Tambyah P (2019). Risk factor profiles and clinical outcomes for children and adults with pneumococcal infections in Singapore: a need to expand vaccination policy?. PLoS One.

[10] Hsieh YC, Lee WS, Shao PL, Chang LY, Huang LM (2008). The transforming Streptococcus pneumoniae in the 21st century. Chang Gung Med J.

[11] McIntosh ED, Reinert RR (2011). Global prevailing and emerging pediatric pneumococcal serotypes. Expert Rev Vaccines.

[12] Kudo D, Kushimoto S, Miyagawa N, Sato T, Hasegawa M, Ito F, Yamanouchi S, Honda H, Andoh K, Furukawa H (2018). The impact of organ dysfunctions on mortality in patients with severe sepsis: a multicenter prospective observational study. J Crit Care.

[13] Lima AP, Beelen P, Bakker J (2002). Use of a peripheral perfusion index derived from the pulse oximetry signal as a noninvasive indicator of perfusion. Crit Care Med.

[14] Diawara I, Barguigua A, Katfy K, Nayme K, Belabbes H, Timinouni M, Zerouali K, Elmdaghri N (2017). Molecular characterization of penicillin non-susceptible Streptococcus pneumoniae isolated before and after pneumococcal conjugate vaccine implementation in Casablanca, Morocco. Ann Clin Microbiol Antimicrob.

[15] Sorensen UB (1993). Typing of pneumococci by using 12 pooled antisera. J Clin Microbiol.

[16] Bechini A, Boccalini S, Bonanni P (2009). Immunization with the 7-valent conjugate pneumococcal vaccine: impact evaluation, continuing surveillance and future perspectives. Vaccine.

[17] Montgomery JM, Lehmann D, Smith T, Michael A, Joseph B, Lupiwa T, Coakley C, Spooner V, Best B, Riley ID (1990). Bacterial colonization of the upper respiratory tract and its association with acute lower respiratory tract infections in Highland children of Papua New Guinea. Rev Infect Dis.

[18] Leach AJ, Boswell JB, Asche V, Nienhuys TG, Mathews JD (1994). Bacterial colonization of the nasopharynx predicts very early onset and persistence of otitis media in Australian Aboriginal infants. Pediatr Infect Dis J.

[19] Faden H, Duffy L, Wasielewski R, Wolf J, Krystofik D, Tung Y (1997). Relationship between nasopharyngeal colonization and the development of otitis media in children. Tonawanda/Williamsville pediatrics. J Infect Dis.

[20] Backhaus E, Berg S, Andersson R, Ockborn G, Malmstrom P, Dahl M, Nasic S, Trollfors B (2016). Epidemiology of invasive pneumococcal infections: manifestations, incidence and case fatality rate correlated to age, gender and risk factors. BMC Infect Dis.

[21] van den Heuvel D, Jansen MAE, Nasserinejad K, Dik WA, van Lochem EG, Bakker-Jonges LE, Bouallouch-Charif H, Jaddoe VWV, Hooijkaas H, van Dongen JJM (2017). Effects of nongenetic factors on immune cell dynamics in early childhood: the generation R study. J Allergy Clin Immunol.

[22] Radetsky M (2014). Fulminant bacterial meningitis. Pediatr Infect Dis J.

[23] Kastenbauer S, Pfister HW (2003). Pneumococcal meningitis in adults: spectrum of complications and prognostic factors in a series of 87 cases. Brain.

[24] Mook-Kanamori BB, Geldhoff M, van der Poll T, van de Beek D (2011). Pathogenesis and pathophysiology of pneumococcal meningitis. Clin Microbiol Rev.

[25] Brouwer MC, McIntyre P, Prasad K, van de Beek D. Corticosteroids for acute bacterial meningitis. Cochrane Database Syst Rev. 2015;2015(9):CD004405. 10.1002/14651858.CD004405.pub5 PMC6491272 26362566

[26] Barlam TF, Morgan JR, Kaplan WA, Outterson K, Pelton SI (2019). Disproportionate exposure to antibiotics in children at risk for invasive pneumococcal disease: potential for emerging resistance and opportunity for antibiotic stewardship. J Pediatr Infect Dis Soc.

[27] Tin Tin Htar M, Christopoulou D, Schmitt HJ (2015). Pneumococcal serotype evolution in Western Europe. BMC Infect Dis.

[28] Subspecialty Group of Respiratory Diseases TSoPCMA, Editorial Board CJoP: [Guidelines for management of community acquired pneumonia in children (the revised edition of 2013) (I)]. Zhonghua Er Ke Za Zhi. 2013;51(10):745–752. 24406226

[29] Hung IF, Tantawichien T, Tsai YH, Patil S, Zotomayor R (2013). Regional epidemiology of invasive pneumococcal disease in Asian adults: epidemiology, disease burden, serotype distribution, and antimicrobial resistance patterns and prevention. Int J Infect Dis.

[30] Qian J, Yao K, Xue L, Xie G, Zheng Y, Wang C, Shang Y, Wang H, Wan L, Liu L (2012). Diversity of pneumococcal surface protein A (PspA) and relation to sequence typing in Streptococcus pneumoniae causing invasive disease in Chinese children. Eur J Clin Microbiol Infect Dis.

[31] Waight PA, Andrews NJ, Ladhani NJ, Sheppard CL, Slack MP, Miller E (2015). Effect of the 13-valent pneumococcal conjugate vaccine on invasive pneumococcal disease in England and Wales 4 years after its introduction: an observational cohort study. Lancet Infect Dis.

[32] Isaacman DJ, McIntosh ED, Reinert RR (2010). Burden of invasive pneumococcal disease and serotype distribution among Streptococcus pneumoniae isolates in young children in Europe: impact of the 7-valent pneumococcal conjugate vaccine and considerations for future conjugate vaccines. Int J Infect Dis.

